# Immunotherapy in Lung Cancer: Current Landscape and Future Directions

**DOI:** 10.3389/fimmu.2022.823618

**Published:** 2022-02-09

**Authors:** Hirva Mamdani, Sandro Matosevic, Ahmed Bilal Khalid, Gregory Durm, Shadia I. Jalal

**Affiliations:** ^1^ Department of Oncology, Barbara Ann Karmanos Cancer Institute, Wayne State University, Detroit, MI, United States; ^2^ Department of Industrial and Physical Pharmacy, Purdue University, West Lafayette, IN, United States; ^3^ Department of Internal Medicine, Indiana University, Indianapolis, IN, United States; ^4^ Department of Internal Medicine, Division of Hematology/Oncology, Indiana University Melvin and Bren Simon Cancer Center, Indiana University School of Medicine, Indianapolis, IN, United States

**Keywords:** immune checkpoint inhibitors, immunotherapy, lung cancer, engineered immune cells, cellular therapy, DNA repair

## Abstract

Over the past decade, lung cancer treatment has undergone a major paradigm shift. A greater understanding of lung cancer biology has led to the development of many effective targeted therapies as well as of immunotherapy. Immune checkpoint inhibitors (ICIs) have shown tremendous benefit in the treatment of non-small cell lung cancer (NSCLC) and are now being used as first-line therapies in metastatic disease, consolidation therapy following chemoradiation in unresectable locally advanced disease, and adjuvant therapy following surgical resection and chemotherapy in resectable disease. Despite these benefits, predicting who will respond to ICIs has proven to be difficult and there remains a need to discover new predictive immunotherapy biomarkers. Furthermore, resistance to ICIs in lung cancer is frequent either because of a lack of response or disease progression after an initial response. The utility of ICIs in the treatment of small cell lung cancer (SCLC) remains limited to first-line treatment of extensive stage disease in combination with chemotherapy with modest impact on overall survival. It is thus important to explore and exploit additional targets to reap the full benefits of immunotherapy in the treatment of lung cancer. Here, we will summarize the current state of immunotherapy in lung cancer, discuss novel targets, and explore the intersection between DNA repair defects and immunotherapy.

## Introduction

Lung cancer is the number one cause of cancer related mortality. Over the last decade, a greater understanding of the biology of lung cancer at the molecular level has led to the development of new and effective therapies resulting in improvement in overall survival (OS), mostly driven by advances in the treatment of non-small cell lung cancer (NSCLC) (1). Traditionally, advanced NSCLC was thought to be one disease. More recently, it has been recognized that NSCLC is on a biological level a set of multiple diseases. Targeted treatments are standard of care in driver mutation positive NSCLC with numerous approved targeted therapies (2). In patients without a driver mutation, immunotherapy in the form of immune checkpoint inhibitor(s) (ICIs) is currently an integral part of the treatment (2). Programmed cell death ligand-1 (PD-L1) is one of the predictive biomarkers of response to checkpoint inhibitors. PD-L1 expression is an imperfect biomarker but is the most robust clinical one. NSCLC, however, continues to have a high mortality rate. Most patients with advanced disease eventually progress on first-line treatment and second-line options are limited for patients without a targetable mutation. Moreover, the clinical application of ICI in the treatment of small cell lung cancer (SCLC) has significantly lagged behind that of NSCLC. There remains a critical need to further harness the power of the immune system, expand treatment options, and delay resistance to ICIs. In this article, we will discuss the current standard immunotherapy treatments and potential novel immunotherapy targets and approaches in lung cancer. We will, in addition, shed light on the interplay between DNA repair defects and immunotherapy, an area of great research interest.

## Current Role of Immunotherapy in Lung Cancer

The relentless nature of any cancer is often attributed to its vast mutational repertoire equipping the cancer cells with mechanisms to develop resistance to commonly used treatment strategies. It is not surprising that lung cancer, with its major histologic subtypes, is among the top five tumor types carrying the highest number of somatic mutations (3). In the first decade of 21^st^ century, median OS of patients diagnosed with advanced NSCLC and SCLC was one year. The discovery of actionable driver genomic alterations and development of targeted therapies led to striking improvement in OS of a subset of NSCLC patients. The survival of the vast majority of patients with NSCLC without an actionable genomic driver and virtually all patients with SCLC remained limited and platinum-based chemotherapy was the mainstay of first-line therapy for these patients.

The discovery of immune checkpoints and subsequent development of Nobel Prize winning ICIs have brought in a radical revolution in the therapeutic landscape of lung cancer, specifically NSCLC (4, 5). Of the several known immune checkpoints utilized by the tumor to evade host immune system, the best known and farthest along in clinical application is programmed cell death protein-1/programmed cell death ligand-1(PD-1/PD-L1) and cytotoxic T lymphocyte antigen-4 (CTLA-4) pathways. Inhibition of these pathways enables priming and anti-tumor activity of cytotoxic T-cells, the essential steps that are otherwise inhibited by the expression of B7-1/2 and PD-L1 by the antigen presenting cells carrying tumor associated antigens and tumor cells, respectively (4).

The first breakthrough in the utility of ICIs in treatment of lung cancer was in the form of PD-1 inhibitor nivolumab as second-line therapy for patients with advanced NSCLC, when randomized phase III trials showed superior objective response rate (ORR) and OS with nivolumab compared to docetaxel in patients with advanced squamous and non-squamous NSCLC following progression on platinum-based chemotherapy (6, 7). Shortly thereafter, another PD-1 inhibitor pembrolizumab and PD-L1 inhibitor atezolizumab were approved by the US FDA for the same indication, based on superior efficacy of these agents compared to docetaxel in second-line setting (8, 9). The success of ICIs in second-line setting paved the way for their use in first-line treatment of advanced NSCLC. A plethora of phase III clinical trials reported over the past five years, showing durable responses and unprecedented improvement in OS with ICI or ICI plus platinum-based chemotherapy compared to chemotherapy alone, have rapidly expanded first-line treatment options for patients with advanced NSCLC not harboring sensitizing EGFR mutations or ALK translocations. These options include pembrolizumab, atezolizumab, cemiplimab, nivolumab plus CTLA-4 inhibitor ipilimumab, pembrolizumab plus platinum-based chemotherapy, atezolizumab plus platinum-based chemotherapy with or without bevacizumab (for non-squamous histology), and nivolumab plus ipilimumab plus two cycles of platinum-based chemotherapy (10–17). The choice of therapy in clinical practice is largely determined by PD-L1 expression, burden of disease, and tumor mutation profile. Besides the improvement in response rates and OS, one of the most fascinating aspects of using ICI-based therapies in NSCLC is the durability of survival benefit. For example, recently reported 5-year outcomes of landmark KEYNOTE-024 trial comparing pembrolizumab with chemotherapy as first-line treatment for patients with advanced NSCLC harboring PD-L1 expression of ≥50% demonstrated unprecedented 5-year OS of 32% with pembrolizumab (18). Randomized trials comparing nivolumab with docetaxel in second-line treatment of advanced NSCLC have also reported that a subset of patient derive prolonged and clinically meaningful survival benefit with ICI (19).

The success march of ICI in NSCLC has expanded to unresectable stage III and more recently to resectable stage II-IIIA disease. In a randomized phase III trial comparing PD-L1 inhibitor durvalumab with placebo in patients with unresectable stage III NSCLC who had non-progressive disease following concurrent chemoradiation, durvalumab showed superior progression free survival (PFS) and OS which were sustained at 5-year follow up, further affirming the durability of anti-tumor activity of ICI in NSCLC (20, 21). Another phase III trial comparing atezolizumab with best supportive care in patients with resectable stable IB-IIIA NSCLC following complete surgical resection and adjuvant platinum-based chemotherapy showed superior disease-free survival (DFS) with atezolizumab which led to recent FDA approval of the agent for adjuvant therapy for patients with resected stage II-IIIA disease with positive PD-L1 expression (22).

In contrast to the exponential growth of the ICI field in NSCLC, the success in SCLC remains disappointing. The only breakthrough in the treatment of extensive-stage SCLC over the past three decades has been the addition of PD-L1 inhibitors durvalumab or atezolizumab to platinum-based chemotherapy for first-line treatment. While the combination has now become the standard of care, the improvement in median OS with the addition of ICI to chemotherapy is modest at best (23, 24). The utility of ICI therapy, either concurrently or sequentially following chemoradiation, in patients with limited-stage SCLC is being evaluated in clinical trials (ClinicalTrials.gov identifiers: NCT03811002, NCT03703297), although a recently reported randomized phase II trial showed no improvement in PFS with nivolumab and ipilimumab following chemoradiation in this setting (25).

## Biomarkers of Response and Resistance to Immunotherapy in Lung Cancer

Despite the increasing role of immunotherapy and specifically PD-1/PD-L1 checkpoint inhibition in lung cancer, a substantial number of patients do not benefit from these therapies. In addition, immune-related toxicities occur in a subset of patients diminishing quality of life, adding to healthcare costs, and resulting in severe impairment or death (26, 27). Considering this, it is imperative that clinically useful predictive biomarkers be developed to appropriately choose those patients most likely to benefit and to avoid those with a small chance of efficacy and/or increased risk of toxicity.

### PD-L1 Expression

With the exception of genomic driver mutations, PD-L1 is the only biomarker recommended by the National Comprehensive Cancer Network (NCCN) guidelines to aid in making treatment decisions in metastatic NSCLC (2). Multiple studies have demonstrated the predictive capability of this biomarker in stage IV NSCLC (8, 10, 28). Nearly all these trials have suggested not only that PD-L1 expression levels can help with patient selection, but also, that the degree of immunotherapy benefit can be predicted by the magnitude of PD-L1 expression (9, 14, 29, 30). In contrast, there are several trials which dispute the benefit of PD-L1 as a viable biomarker for predicting response to PD-1/PD-L1 checkpoint inhibitors (6, 7, 31).

The predictive role of PD-L1 expression in the non-metastatic setting is gradually transpiring. In PACIFIC trial, PD-L1 negative patients exhibited less robust PFS and OS compared to PD-L1 positive patients following treatment with durvalumab (32). In contrast to this, correlative analysis of a smaller phase II trial of consolidation pembrolizumab after chemoradiation showed no difference in outcomes for PD-L1 positive versus negative patients (33). In the adjuvant setting, DFS was improved with atezolizumab following surgery and adjuvant chemotherapy only in the PDL1≥1% group, with the PD-L1 ≥50% group exhibiting the largest benefit (22).

### Tumor Mutational Burden (TMB)

TMB is defined as the total number of mutations per megabase (Mb) of exonic regions of evaluated genes in a tumor specimen. As the number of mutations increases, the potential number of new transcribed proteins and neoantigens also goes up. This increase in neoantigen presence is hypothesized to enhance tumor immunogenicity and improve the likelihood that patients respond to checkpoint inhibition. Tissue and blood based TMB have been studied in several lung cancer and tumor agnostic studies and have been shown to predict benefit from various ICI (9, 13, 30, 31). More recently, the FDA has approved pembrolizumab for patients with any tumor type that demonstrates a high TMB (≥10 mut/Mb) following results of the KEYNOTE-158 trial (34). Studies evaluating the combination of PD-L1 and TMB as a composite biomarker have suggested improved predictive capability for the combination (31, 35).

There are no clinically useful biomarkers to help guide the use of ICIs in extensive stage SCLC. Despite presenting with immune-mediated paraneoplastic syndromes and often exhibiting high TMB, SCLC does not typically demonstrate the same clinical benefit to checkpoint inhibition that is seen in NSCLC. The landmark clinical trials establishing the role of ICI in combination with chemotherapy in first-line treatment of extensive-stage SCLC did not demonstrate any discernible difference in response rates or clinical utility of ICI for any PD-L1 subgroup (36, 37).

### Specific Genomic Alterations

There is some data to suggest that specific genomic alterations may predict better or worse responses to ICI. Much of this research has focused on determining genomic markers for immunotherapy resistance. STK11/LKB1 is a distinct subgroup of Kirsten Rat Sarcoma Virus (KRAS)- mutant lung adenocarcinoma. Some studies have suggested that the presence of KRAS mutation is a predictive of superior response rates from ICIs (38). However, specific co-mutations such as STK11/LKB1 have demonstrated poor immunogenic responses. In two separate lung adenocarcinoma cohorts, patients with KRAS and STK11/LKB1 co-mutations showed lower ORR compared with KRAS mutation alone and one of the cohorts noted a significantly lower PFS and OS during immunotherapy treatment (39). A second retrospective trial of patients with STK11 alone and STK11/KRAS co-mutations treated with immunotherapy showed poorer OS and PFS for those with STK11 mutations versus their STK11-wildtype counterparts (40). However, this study also notes poorer outcomes for STK11-mutant patients when treated with chemotherapy, and an additional analysis suggests that poor outcomes in this population are prognostic rather than predictive of poor immune response (41)

Data has also been presented for ICI response in patients with common ‘targetable’ genomic alterations such as EGFR mutations and ALK fusions. Retrospective studies have demonstrated very low response rates with ICI in EGFR mutant NSCLC compared to EGFR wildtype tumors (42, 43). Prospective trials of ICI monotherapy in patients with EGFR mutant NSCLC have yielded similarly disappointing results (36, 44). Though the data is more scarce, patients with ALK gene rearrangements also appear to demonstrate poor responses to ICI monotherapy (36, 45).

### Circulating Tumor DNA (ctDNA)

Analysis of any tumor-derived material circulating in the peripheral blood, also called ‘Liquid Biopsy’, is gaining increasing popularity in oncology because of its feasibility and relatively rapid turnaround time. Of the multitude of blood-based biomarker analyses, circulating tumor DNA (ctDNA) is the most commonly used modality (46). The role of ctDNA analysis in the context of ICI therapy in lung cancer is currently limited to detection of specific genomic alterations noted above. Several commercial platforms are offering blood-based TMB assays, however, their clinical utility is yet to be validated in large prospective trials. Early data from studies looking at serial monitoring of ctDNA as a biomarker of response and survival with ICI show promising correlation between molecular and radiographic response (47). With well-designed future studies on a larger scale, it is conceivable that the depth of molecular response will guide the optimum duration of ICI therapy in future, a question that remains largely unanswered at this time.

## Challenges Facing ICI Therapy in Lung Cancer

The unprecedented success of ICIs in the treatment of lung cancer is not without several major challenges. Firstly, nearly 70% of patients with advanced NSCLC and 80% of patients with SCLC do not derive durable benefit from ICI based therapies. Putative mechanisms of inherent or acquired resistance to ICI include T-cell exhaustion, co-expression of inhibitory receptors, altered metabolism through Indoleamine 2, 3-dioxygenase 1 (IDO-1) and increased adenosine production, high copy number loss of tumor suppressor genes, and decreased antigen presentation (48). Second, as noted above, the quest for a perfect and reliable biomarker of response to ICI is still ongoing. While PD-L1 expression is the most widely used biomarker in clinical practice, its predictive value is not absolute. Inter-tumor and intra-tumor heterogeneity of PD-L1 expression, a wide variety of PD-L1 assays and cutoff values utilized to define PD-L1 positivity, and the effect of handling and storage of tumor tissue on PD-L1 analysis render it an imperfect biomarker (11, 49–52). Studies have shown significant discordance in PD-L1 expression between lung biopsies and corresponding resected tumors, between primary and metastatic sites, and among different metastatic sites (53–55). Finally, SCLC remains an invincible enemy with only a limited success achieved with incorporation of currently available ICIs in the treatment paradigm, largely driven by an immunosuppressive tumor microenvironment (TME) (56). These challenges have warranted development of novel approaches to harness the power of the immune system in combating this historically relentless disease.

## Immunotherapy Approaches Beyond PD-1/PD-L1 and CTLA-4 Inhibition in Lung Cancer

### Novel Immune Checkpoint Targets

As noted above, PD-1/PD-L1 and CTLA-4 inhibitors are the most commonly used ICIs in lung cancer, yet development of resistance to these agents remains an insurmountable challenge. In recent years, inhibitors of novel immune checkpoint targets have shown encouraging results in pre-clinical and early clinical studies, potentiating the pursuit of new therapeutic strategies to overcome the resistance to conventional ICIs.

#### T-Cell Immunoreceptor With Ig and ITIM Domains (TIGIT)

TIGIT is a promising new immune checkpoint. It is expressed on activated T cells, natural killer (NK) cells, and regulatory T cells (Tregs). TIGIT binds to two ligands, CD155 (PVR) and CD112 (PVRL2, nectin-2), that are expressed by tumor cells and antigen-presenting cells in the tumor microenvironment (57). Dual PD-1/TIGIT blockade potently increases tumor antigen-specific CD8+ T cell expansion and function *in vitro* and promotes tumor rejection in mouse tumor models (58, 59). In a recently reported randomized phase II trial, combination of anti-TIGIT antibody tiragolumab with atezolizumab led to clinically meaningful improvement in ORR and PFS compared to placebo plus atezolizumab as first-line treatment of patients with advanced, PD-L1 positive NSCLC (60). Based on these results FDA has granted breakthrough therapy designation to tiragolumab in NSCLC. A confirmatory phase III trial is ongoing in this patient population (ClinicalTrials.gov identifier: NCT04294810). Early results from a phase I trial of another anti-TIGIT antibody vibostolimab has shown clinical activity in combination with pembrolizumab in PD-1/PD-L1 naïve and refractory patients with advanced NSCLC (61). An ongoing phase III trial is comparing combination of vibostolimab plus pembrolizumab with pembrolizumab alone in patients with PD-L1 positive advanced NSCLC (ClinicalTrials.gov identifier: NCT04738487).

#### Lymphocyte Activation Gene-3 (LAG-3)

LAG-3 is expressed on activated CD4+ and CD8+ T cells, Tregs, a subpopulation of NK cells, B cells, and plasmacytoid dendritic cells (pDCs). LAG-3 signaling plays a negative regulatory role in T helper 1 (Th1) cell activation, proliferation and cytokine secretion, a function that is exploited by tumor cells to evade the host immune system (62). Among several different LAG-3 inhibitors under development, monoclonal antibody relatlimab, is farthest along in clinical trials. The combination of relatlimab with nivolumab has shown significant improvement in PFS compared to nivolumab alone in patients with melanoma (63). A phase II trial evaluating the efficacy of relatlimab in combination with nivolumab and chemotherapy as first-line treatment of advanced NSCLC is currently ongoing (ClinicalTrials.gov identifier: NCT04623775).

#### T-Cell Immunoglobulin and Mucin-Domain Containing-3 (TIM-3)

TIM-3, a negative regulator of T cell response and also called hepatitis A virus cellular receptor 2 (HAVCR2), expressed on CD4+ and CD8+ T cells, NK cells, DCs, Tregs, monocytes, and macrophages, is another emerging immune checkpoint (64). Higher expression of TIM-3 has been associated with poor prognosis in solid malignancies and inhibition of TIM-3 in combination with PD-1 inhibition has been shown to have anti-tumor activity (65, 66). At least eight different TIM-3 inhibitors are in development and have shown superior efficacy of simultaneous inhibition of TIM-3 pathway and PD-1 pathway over single-agent treatment (67).

#### NK Group 2 Member A (NKG2A)

NKG2A is a cell surface molecule, which is typically expressed by NK cells, but expression can be induced on T cells as well, especially on CD8+ T cells (68). HLA-E, a ligand of NKG2A, expression has been demonstrated to have immunosuppressive function through binding to NKG2A (69). Overexpression of HLA-E on cancer cells has been correlated with poor outcomes (70). Monalizumab, a monoclonal antibody targeting NKG2A, has shown promising anti-tumor activity in early clinical trials in lung cancer, including recently reported interim analysis of a phase II trial showing improved ORR and PFS with monalizumab in combination with durvalumab compared to durvalumab alone in patients with unresectable, Stage III NSCLC who did not progress after concurrent chemoradiation therapy (71).

#### CD73

CD73, an ecto-5′-nucleotidase (NT5E), serves as an immune checkpoint by generating adenosine which suppresses immune activation through the A2A receptor (72). CD73 is upregulated in a variety of tumors including lung cancer, and higher expression of CD73 in the tumor tissue is associated with poor outcomes (72–75). Preclinical studies have established a strong foundation for evaluating CD73 inhibition in combination with PD-1/PD-L1 inhibition by demonstrating synergistic anti-tumor activity through augmentation of intra-tumoral infiltration of CD8+ tumor-specific T cells (76, 77). Recently reported early results of a phase II clinical trial evaluating oleclumab, a monoclonal antibody against CD73, in combination with durvalumab following chemoradiation in locally advanced unresectable stage III NSCLC showed improved PFS with the combination compared to durvalumab alone, with a manageable safety profile (78). A number of trials are underway to evaluate oleclumab containing regimens in lung cancer.

Apart from these, several other immune checkpoint targets are under investigation, including V-domain immunoglobulin suppressor of T cell activation (VISTA), B7-H3 (CD276), IDO-1, glucocorticoid-induced TNFR-related receptor (GITR), and CD47. A multitude of ongoing clinical trials are evaluating inhibitors of these targets by themselves and in combination with PD-1/PD-L1 inhibition (**Table 1**).

**Table 1:** Novel immune checkpoint inhibitors and ongoing clinical trials.

Target	Proposed function	Drug	ClinicalTrials.gov Identifier	Phase
**TIGIT**	Inhibitory receptor on activated T-cells that binds to CD155 and CD112 on tumor cells	Tiragolumab (anti TIGIT mAb)	NCT04256421 NCT04294810 NCT04513925 NCT04619797 NCT04832854 NCT04308785	III III III II II II
		Vibostolimab (anti TIGIT mAb)	NCT04738487 NCT02964013	III I
		Domvanalimab (anti TIGIT mAb)	NCT04736173 NCT04262856 NCT04791839	III II II
**LAG 3**	Inhibitory co-receptor in T-cell stimulation	Eftilagimod alpha (soluble LAG-3 fusion protein)	NCT03625323	II
		Relatlimab (anti LAG3 mAb)	NCT04623775	II
		BI 754111 (anti LAG3 mAb)	NCT03156114	I
**TIM-3**	Inhibitory receptor on CD4+ and CD8+ cells that causes Th1 suppression	RO7121661 (bispecific anti TIM-3/anti-PD1 mAb)	NCT03708328	I
		MGB453 (anti-TIM3-mAb)	NCT02608268	I
		TSR-022 (anti-TIM-3 mAb)	NCT03307785	I
**NKG2A**	Inhibitory receptor on NK and CD8+ T cells	Monalizumab (anti NKG2A mAb)	NCT03833440 NCT03822351 NCT05061550	II II II
**CD73**	Generates adenosine which suppresses immune activation through A2A receptor	Oleclumab (anti CD73 mAb)	NCT05061550 NCT03822351 NCT03334617	II II II
**IDO-1**	Enzyme on tumor cells that metabolizes tryptophan to kynurenine and subsequently inhibits T-cell differentiation	LY3381916 (IDO-1 inhibitor)	NCT03343613	I
		Epacadostat (IDO-1 inhibitor)	NCT03322540 NCT03322566	II II
		Indoximod (IDO-1 inhibitor)	NCT02460367	I
**B7-H3**	Inhibitor of CD4+ and CD8+ cells, decreases IL2 and IFN- γ production	Enoblituzumab (anti B7-H3 mAb)	NCT02381314 NCT02475213	I I
**CD27**	Expressed on T-cells; agonism thought to stimulate effector T-cells and repress T-regs	Varlilumab (anti-CD27 mAb)	NCT01460134 NCT04081688	I I
**GITR**	GITR receptor binding with GITR-ligand promotes tumor cell apoptosis and increased activity of T-cells and NK cells	MEDI1873 (GITRL/IgG1 agonist fusion protein)	NCT02583165	I
		BMS-986156 (IgG1 agonist mAb to GITR)	NCT04021043	I/II
**CD47 and SIRPα**	CD47 (overexpressed on tumor cells) binds to SIRPα receptor on myeloid cells and restricts phagocytosis	TTI-621(soluble recombinant fusion protein against CD47)	NCT02663518	I
		Magrolimab (anti CD47 mAb)	NCT04827576	II
**VISTA**	Homologous to PD-L1; suppresses T-cell proliferation	CA-170 (oral PD-L1, PD-L2 and VISTA checkpoint antagonist)	NCT02812875	I

TIGIT, T cell immunoreceptor with Ig and ITIM domains; mAb, monoclonal antibody; LAG-3, Lymphocyte activation gene 3; TIM-3, T-cell immunoglobulin and mucin-domain containing-3; NKG2A, NK group 2 member A; CD73, Cluster of differentiation 73; IDO1, Indoleamine 2, 3-dioxygenase 1; B7-H3, B7 homolog 3; IFN- γ, Interferon Gamma; CD27, Cluster of differentiation 27; GITR, glucocorticoid-induced TNFR-related receptor; CD47 and SIRPα, Cluster of differentiation 47 and Signal Regulatory Protein Alpha; VISTA, V-domain immunoglobulin suppressor of T cell activation; IL-2, Interleukin 2; T-regs, Regulatory T-cells; NK cells, Natural Killer Cells; PD-L1, Programmed Death-Ligand 1.

### Combination with Conventional Therapeutic Strategies

#### Chemotherapy

As noted above, chemotherapy in combination with ICI has shown superiority over chemotherapy alone in several randomized controlled trials in NSCLC and SCLC. In these trials, patients typically received 4 cycles of platinum-based chemotherapy in combination with ICI followed by maintenance ICI alone or pemetrexed plus ICI in case of non-squamous NSCLC. Subsequently, CheckMate-9LA trial demonstrated feasibility of using shorter duration, i.e. 2 cycles, of chemotherapy in combination with PD-1 and CTLA-4 inhibitors without comprising the efficacy of the regimen (17). While the synergistic activity of ICIs in combination with chemotherapy is well established and that the addition of ICI may allow for shorter duration of chemotherapy, several questions remain unanswered, including the optimum number of chemotherapy cycles in the initial induction phase, whether pemetrexed continuation is critical in the maintenance phase for non-squamous NSCLC, predictive biomarkers to inform decision making with regards to the duration of chemoimmunotherapy, and value of adding ICI to second-line chemotherapy following progression on first-line chemoimmunotherapy.

#### Radiation

The immunomodulatory effects of radiation are well established and include a shift in tumor associated macrophage polarization, activation of tumor-associated dendritic cells, improved T-cell homing to tumors, destruction of immunosuppressive stromal cells within the TME, and induction of immunogenic cell death (79). Consequently, combining ICI with radiation, either concurrently or sequentially, has been an area of great interest. A number of clinical trials have been initiated to build on the success of PACIFIC trial with consolidation durvalumab following chemoradiation in patients with locally advanced unresectable NSCLC. A notable concern with combining ICI with radiation is the development of toxicity, specifically pneumonitis. In a phase II trial of pembrolizumab with concurrent chemoradiation, the rate of grade ≥ 3 pneumonitis was 8% compared to 3.4% reported in PACIFIC trial (80). Future approaches directed towards modifying the dosing and schedule of radiation when used in close proximity with ICI may enhance the feasibility and minimize toxicity of this approach.

#### Targeted Therapy

The list of actionable genomic alterations in NSCLC and corresponding targeted therapeutic approaches have expanded considerably in the recent years. Retrospective studies and subset analyses of prospective trials have shown limited efficacy of ICIs in patients with NSCLC harboring targetable driver alterations (43, 81). Combination of ICIs with EGFR and ALK tyrosine kinase inhibitors has shown increase in severe treatment related toxicity, including pneumonitis and liver dysfunction, with no added clinical activity (82). KRAS mutation is perhaps an exception to this limitation. KRAS G12C inhibitors have shown development of pro-inflammatory TME and durable responses alone as well as in combination with ICIs in mice models (83). Clinical trials combining ICIs with currently available KRAS G12C inhibitors, sotorasib and adagrasib, are ongoing (ClinicalTrials.gov identifiers: NCT04185883, NCT04613596).

### Combination With DNA Repair Targeting Agents

DNA damage is a hallmark of lung cancer and is most apparent in smoking induced NSCLC and SCLC (84). Smoking induced DNA damage triggers several DNA response pathways (85). Accurate and faithful DNA replication is critical for maintenance of genomic stability in all cellular divisions including that of immune cells (86, 87). While traditionally DNA repair has been studied in the context of sensitivity to platinum-based chemotherapy and PARP inhibition, mounting evidence suggests the role of DNA repair defects in predicting response to ICIs in a variety of tumors, including NSCLC (88–91). A well-known example is the efficacy of pembrolizumab in mismatch repair deficient tumors irrespective of PD-L1 expression (92). Germline mutations in *BRCA2* or *POLE* have also been connected to increased ICI sensitivity (93, 94). A retrospective study has shown strong association of somatic *BRCA1*, *PALB2*, and *POLE* mutations with high TMB in NSCLC (95). The precise mechanisms behind the association of DNA repair defects and response to ICI, however, remain poorly understood. Genomic instability resulting from these defects, higher neoantigen load and TMB, and association with activation of cGAS-STING pathway are among the postulated key players (90, 96).

In addition to utilizing DNA repair defects in predicting response to ICI in lung cancer, an increasing amount of research is focusing on the prospect of combining ICIs with DNA repair targeting agents in NSCLC. Perhaps the farthest along in this race are PARP inhibitors and ATR inhibitors. Preclinical studies have shown upregulation of PD-L1 expression with PARP inhibition, re-sensitization of PARP inhibitor treated cancer cells to T-cell mediated killing through PD-L1 blockade, and enhanced *in vivo* therapeutic efficacy of PARP inhibition in combination with anti-PD-L1 therapy (97). Three large ongoing clinical trials are evaluating the efficacy and safety of combination of ICI with PARP inhibitor in NSCLC, including KEYLINK-006 (ClinicalTrials.gov identifier: NCT03976323), KEYLINK-008 (ClinicalTrials.gov identifier: NCT03976362), and ORION (ClinicalTrials.gov identifier: NCT03775486). Similarly, a trial of ATR inhibitor ceralasertib evaluating its combination with paclitaxel in patients with a variety of solid organ malignancies showed upregulation of PD-L1 expression in paired tumor samples following treatment with ceralasertib (98). As a result of this initial finding, an ongoing study is evaluating combination of ceralasertib with durvalumab in patients with advanced NSCLC following progression on ICI therapy (ClinicalTrials.gov Identifier: NCT03334617). While these studies are being conducted in all NSCLC patients, it remains unclear whether the benefit of such combinations will be restricted to those with DNA repair defective tumors. Additionally, clinically feasible and reproducible biomarkers of DNA repair defects in lung cancer will need to be evaluated and validated in prospective studies.

**Figure 1:**
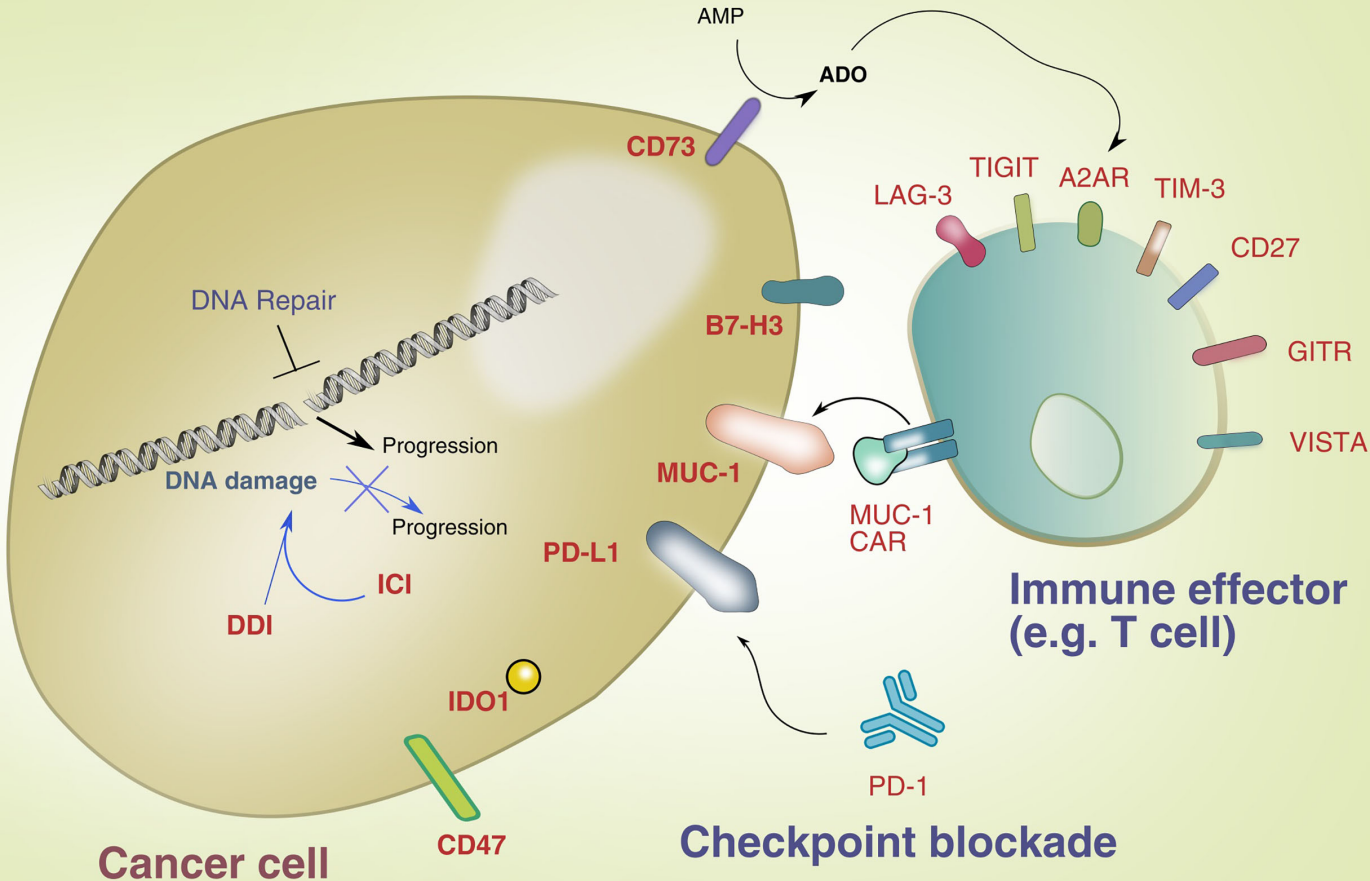
Novel immunotherapy targets and intersection with DNA repair.

**Figure 1** illustrates novel immunotherapy targets and intersection with DNA repair in lung cancer.

### Cellular Therapy in Lung Cancer

Immune cell-based therapy has recently emerged as a promising immunotherapeutic approach to target lung cancer (99). Building on successes in other tumors, immune cells have been exploited for their innate ability to eliminate cancer cells and mount powerful immune responses by recruiting other cells in the TME. To enhance specificity of immune recognition of cancer cells, genetic engineering of T- or NK cells has enabled these cells to target specific antigens expressed on lung cancer cells and reprogram the immune cells’ behavior toward enhanced function. Most of the studies on immune cell-based targeting of lung cancer have been reported for CAR-T cells, though a growing body of work is exploiting the allogeneic nature of NK cells as potentially safer alternatives to infused CAR-T cells (100).

Engineered cell-based therapies for lung cancer have so far been designed to target epidermal growth factor receptor (EGFR) and variant III (EGFRviii), glypican 3 (GPC3), human epidermal growth factor receptor 2 (HER2), Protein tyrosine kinase 7 (PTK7), erythropoietin-producing hepatocellular carcinoma A2 (EphA2), mesothelin (MSLN), prostate stem cell antigen (PSCA), mucin 1 (MUC1), carcinoembryonic antigen (CEA), natural killer group 2D (NKG2D), tyrosine kinase-like orphan receptor 1 (ROR1), and programmed cell death ligand 1 (PD-L1),lung-specific X (LunX), and delta-like 3 (DLL3) (101–104).

Future clinical development of cellular therapies targeting many of these antigens has been challenged by toxicities observed upon infusion of CAR-T products. For instance, infusion of ERBB2-specific CAR-T cells to treat a patient with colon cancer with metastasis to lungs and liver caused respiratory distress within 15 minutes of cell infusion (105). The authors of the study speculated that this was due to the high activity of CAR-T cells in lungs following recognition of low levels of ERBB2 expression on lung epithelial cells, thus triggering intense, and toxic, cytokine production. Acute respiratory toxicities were also observed in a cohort of patients treated with CEACAM-5-directed CAR-T cells, again due to expression of CEACAM-5 on the lung epithelium (106). This was accompanied by high levels of IFN-γ and IL-6. This lack of specificity for tumor expressed antigens is one of the major challenges to the clinical development of CAR-T cell therapies in solid tumors, specifically lung cancer.

Though the ideal antigen is one that is exclusively present on tumor cells and absent in healthy tissue, targeting approaches often must grapple with sub-optimal expression of targetable antigens due to their presence in surrounding healthy tissues. In addition to the non-specific nature of expression of many of the targetable antigens in lung cancer, antigen density is highly heterogeneous in the tumors, and their high genomic instability leads to antigen loss or outgrowth variants that can occur in response to persistence targeting by antigen-specific CAR-T cells (107). Antigen loss has spurred the development of engineering strategies that can induce genetic circuitry to immune cells and avoid antigen escape. These include dual or multispecific CAR-T cells, CAR-T cell secreting antibodies such as PD-L1, as well as trigger-responsive CAR-T cells (e.g. synNotch CAR-T cells, which are designed to target multiple antigens and enhance their specificity against the tumor (108, 109).

Despite the demonstrated successes with cell-based therapies with hematological malignancies, the response rates in solid tumors, including lung cancer, have been underwhelming. In lung cancer, the TME presents a complex barrier to the activity of immune cells which often results in resistance to treatment. The homing of CAR-T cells is poor due to several factors acting against them in the lung cancer TME. These include inadequate or mismatched chemokine-chemokine receptor pairs, downregulation of adhesion molecules, aberrant vasculature, unfavorable extracellular matrix (ECM) composition, and immunometabolically adverse conditions such as hypoxia and the presence of immunosuppressive soluble metabolites and factors such as TGF-β, lactate and adenosine. In particular, in lung cancer, the structure of the ECM, composed of collagens, proteoglycans and glycosaminoglycans, has been reported as playing a significant role in the ability of immune cells to successfully home to lung tumors (110). In addition, dysfunction of infiltrating immune cells occurs due to unfavorable immunometabolic conditions in the tumor. Dysfunction, in the case of CAR-T cells, can manifest as exhaustion, senescence, or anergy (111). This results in inadequate immune responses and has triggered the development of “exhaustion-resistant” CAR-T cells, which are engineered by knocking out genes that contribute to T cell dysfunction, such as, in one case, TCR, HLA class I, PD-1, and CTLA-4 using one-shot CRISPR (112). Recent studies have also shown that blocking the adenosine pathway by inhibiting activity of CD73 can enhance immune cell activity and infiltration into lung cancer in preclinical mouse models (113). In these studies, NK cells engineered to target NKG2D on lung cancer were combined with antibody-mediated blockade of CD73 and resulted in deeper intratumoral infiltration and killing ability of these cells.

Finally, CAR-T cells themselves can cause toxicities upon systemic administration. Severe and sometimes lethal cytokine levels have been measured in patients treated with CAR T cells in many clinical trials, not exclusive to lung cancer (114). Mitigation measures include antibody therapy to reduce the burden of cytokine release syndrome, engineering safety switch-based CAR-T cells, or using NK cells. Local administration of immune cell therapies is currently under preclinical and clinical investigation as an approach to directly drive cells to the tumor.

## Conclusion

The landscape of immunotherapy in lung cancer is rapidly expanding and ICIs have become the standard of care treatment for patients with metastatic, locally advanced, and resectable NSCLC with remarkable improvement in OS. The clinical utility of ICI in SCLC is limited to first-line therapy of extensive-stage disease with small improvement in OS. Resistance to immunotherapy, either inherent or acquired, is a major challenge facing the Oncology community. Cellular therapy is a promising and potent addition to the arsenal of immunotherapies for lung cancer. Lack of tumor-specific antigens, hostile TME, and toxicity make cellular therapy an exciting, but undeniably challenging, proposition. Development of novel treatment strategies, including combination and sequencing of PD-1/PD-L1 inhibitors with other ICIs and DNA repair targeting agents, are being evaluated in clinical trials.

## Author Contributions

All authors listed have made a substantial, direct, and intellectual contribution to the work, and approved it for publication.

## Conflict of Interest

The authors have following conflicts of interest within the past 2 years, none of which influenced the work being submitted. HM: Advisory role – Zentalis. GD: Research grant - BMS, AstraZeneca, Merck; Honoraria - AstraZeneca, Curio Science. SJ: Research grant - AstraZeneca, Astex, Tesaro; Consultant – Adaptimmune.

The remaining authors declare that the research was conducted in the absence of any commercial or financial relationships that could be construed as a potential conflict of interest.

## Publisher’s Note

All claims expressed in this article are solely those of the authors and do not necessarily represent those of their affiliated organizations, or those of the publisher, the editors and the reviewers. Any product that may be evaluated in this article, or claim that may be made by its manufacturer, is not guaranteed or endorsed by the publisher.
